# A study of HPV self-sampling by female residents in communities of Zhengzhou, Henan Province, China: a cross-sectional observational study

**DOI:** 10.3389/fpubh.2025.1557678

**Published:** 2025-07-01

**Authors:** Zhe Yang, Yue Lu, Yiping Zhang, Yunfeng Zhang, Jingyi Mu, Yicheng Li, Shihao Mei, Yuru Guo, Wanyue Zhang, Peiru Zhu, Yongjie Li, Guiqin Chen, Ruijiao Zhao, Aixia Hu, Rujia Fan, Yue Wang

**Affiliations:** ^1^Department of Obstetrics and Gynecology, Henan Provincial People's Hospital, Zhengzhou, China; ^2^Department of Obstetrics and Gynecology, People's Hospital of Zhengzhou University, Zhengzhou, China; ^3^Department of Obstetrics and Gynecology, People's Hospital of Henan University, Zhengzhou, China; ^4^Henan International Joint Laboratory of Early Diagnosis and Treatment of Gynecological Malignant Tumors, Zhengzhou, China

**Keywords:** cervical cancer, HPV self-sampling, communities, early screening, HPV typing and quantitative

## Abstract

**Objective:**

Evaluate the feasibility of HPV self-sampling typing and quantitative detection as a cervical cancer screening scheme, and provide new methods to reduce the incidence of cervical cancer.

**Methods:**

This was a cross-sectional observational study of 1,228 female residents in communities in Zhengzhou city, Henan Province, who participated in HPV self-sampling detection. All the samples were subjected to HPV typing and quantitative detection. HPV-positive individuals were recalled for further cervical liquid-based cytology and colposcopy.

**Results:**

The results of this study revealed that 33.71% of female residents lacked awareness of cervical cancer screening. Older age and low educational level are independent influencing factors for the lack of screening awareness. The overall positive rate of HPV was 18.89%. The three most common subtypes were types 16 (2.61%), 52 (2.44%), and 53 (2.28%). Results revealed that age at first sexual intercourse, sexual frequency, parity, having multiple sexual partners, cleaning behavior after sexual intercourse, and using condoms during sexual intercourse were factors related to HPV infection (*P* < 0.05). Logistic regression analysis revealed that age at first sexual intercourse ≤20 years, sexual frequency >2 times/week, parity >1 time, and having multiple sexual partners were risk factors for HPV infection (OR > 1, *P* < 0.05). Frequent cleaning after sexual intercourse and frequent use of condoms during sexual intercourse were protective factors against HPV infection (OR < 1, *P* < 0.05).

**Conclusion:**

HPV self-sampling detection had a good experience and a high degree of acceptance, which can be promoted and applied in cervical cancer screening in Henan province.

## 1 Introduction

Cervical cancer is a malignant neoplasm that develops in the cervix and is the fourth most common tumor among women in the world, seriously threatening women's health. It causes 600,000 cases and 340,000 deaths worldwide each year, 80% of which occur in developing countries (1, 2). More than 99% of cervical cancer cases are caused by human papilloma virus (HPV) infection, and ~90% of patients with HPV infection will automatically clear the virus through the body's own immune system within 1–2 years (3). Only persistent high-risk HPV infection will cause precancerous lesions in the early stage, progressing to cervical cancer after 10–20 years.

At present, more than 200 subtypes have been identified and fully characterized (4). HPV is usually classified into two types: “low-risk” and “high- risk” (5). The high-risk types mainly include HPV16, 18, 31, 33, 35, 39, 45, 51, 52, 56, 58, and 59 (6). The guidelines of the China Food and Drug Administration (CFDA) in 2015 have specified the subtypes of high-risk and medium-risk for HPV detection. The infection rate of high-risk types in various countries and regions is significantly higher than that of low-risk types. However, there are few studies on the analysis of the characteristics of the infected population between high-risk and low-risk types. HPV16 and HPV18 are the two most common types and together cause most cases of cervical cancer and its precursors (7, 8). Globally, 70% of cervical cancer cases are caused by high-risk HPV16 and 18 infections (9). Single infection refers to only a single type of HPV subtype is infected. Multiple infection refers to a host carries multiple HPV subtypes simultaneously. Compared with single infection, it may have different impacts on the development of cervical lesions (10, 11).

In China, ~70%−80% of women are infected with HPV at least once in their life (12). Cervical cancer screening is an effective way to achieve the goals of “early detection, early diagnosis and early treatment” and reduce morbidity and mortality (13). With the increasing prevalence of cervical cancer screening, the global incidence of cervical cancer has significantly decreased (14), so early screening is very important for reducing the incidence and mortality of cervical cancer.

Current screening methods for cervical cancer include cytological screening and HPV testing. Pap smear staining is the standard cytological screening method for detecting cervical cancer (15). Cervical cancer screening through Pap smear staining and microscopic examination can significantly reduce the incidence and mortality rates of cervical cancer. Regular Pap smear staining can help detect changes in disease status early and prompt timely treatment and follow-up. However, Pap smear staining susceptible to human factors such as sampling, smearing, and staining, which result in low sensitivity and high false-negative rates (16). Improved liquid-based cytology has become a widely used method for cervical cancer screening. Eliminating errors caused by human factors in Pap smear staining can improve the quality and accuracy of cervical cytology testing. Compared with traditional Pap smear staining, improved liquid-based cytology has greater sensitivity and specificity, but false positives are more common (17).

Compared with cytological screening, HPV testing is more effective in preventing future cervical lesions (15). The World Health Organization (WHO) recommends that women undergo cervical cancer screening every 3–5 years on the basis of age and risk factors (18). In many developed countries, cervical cancer screening programs have significantly reduced the incidence and mortality rates of cervical cancer. However, as a developing country with a large population and vast territory, China has a lower overall cervical cancer screening rate, and there are issues of low coverage and efficiency in cervical cancer screening. Therefore, strengthening the convenience and feasibility of HPV detection technology and achieving greater coverage are particularly urgent and important, especially in remote and economically underdeveloped areas.

HPV self-sampling, as a new method of HPV testing, allows women to collect specimens themselves in comfortable and convenient locations without the assistance of a healthcare professional; this method has the advantages of ensuring privacy, convenience and efficiency. As a new model of cervical cancer screening, HPV self-sampling has great potential in expanding the scope of screening, simplifying the testing process, and reducing costs. This not only increases the motivation of women for HPV testing, especially those who never have undergone HPV screening but also facilitates coverage in remote and less accessible areas, thereby increasing the coverage of cervical cancer screening and better preventing cervical cancer.

This study tested HPV types and loads from self-samples, investigated HPV infection status and acceptance of self-sampling. Then assessed the feasibility of HPV self-sampling for cervical cancer screening programs, to increase the coverage of cervical cancer screening and offer a new way to reduce the incidence of cervical cancer.

## 2 Method

This is an observational study and approved by the Ethics Committee of Henan Provincial People's Hospital [Approval No: (2021) No. 65]. A total of 1,228 women screened in Zhengzhou, Henan Province, participated in HPV self-sampling and questionnaire surveys between March 2021 and March 2024. All participants in the screening were voluntary, fully understood the investigation content and signed informed consent forms.

### 2.1 Questionnaires used

To protect the privacy of the participants and ensure the authenticity and validity of the survey results, the investigators conducted a one-to-one questionnaire survey on the women who participated in the HPV self-sampling. The questionnaires included three parts: the first part recorded personal information, including age, educational level, smoking status, number of pregnancies, number of deliveries, personal hygiene habits, age at first sexual intercourse, multiple sexual partners, HPV vaccination history; the second part understood the perceptions of cervical cancer, including whether the participants were aware of cervical cancer, knew about HPV, were aware of HPV self-sampling testing, the testing of HPV and ThinPrep^®^ Cytologic Test (TCT) was performed before; the third part assessed the evaluation of self-sampling, including the duration and experience of self-sampling, bleeding after self-sampling, the attitude toward the accuracy of self-sampling results, whether the participants will to accept self-sampling again, etc.; and the investigator assigned a score on the basis of the response of the participants, the score of “no” was 0, and the score of “yes” was 1, a total score ≤ 1 was considered “lack of cognition”. The contents of the paper questionnaires were double entered, and the data were randomly selected for information detection to ensure the authenticity and accuracy of the results.

### 2.2 Recruitment

**Figure 1:**
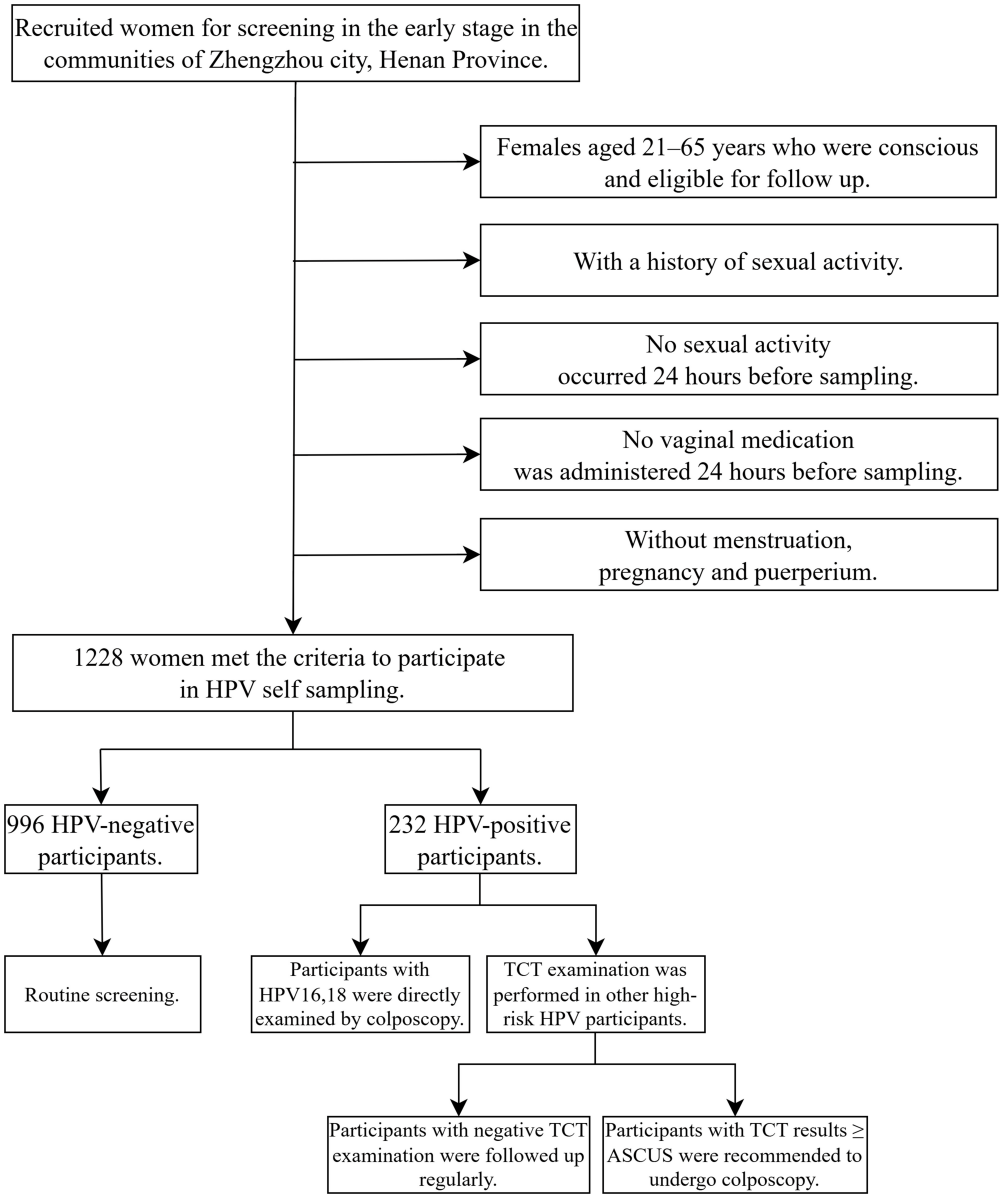
The HPV self-sampling screening and triage plans.

Gynecologists from Henan Provincial People's Hospital and community leaders set up a screening team, released screening information and recruited women for screening in the early stage. According to the inclusion and exclusion criteria, a total of 1,228 volunteers were finally enrolled. All participants received instructions from gynecologists before sampling and completed a questionnaire in a private space after sampling. The specific screening and triage plan as follows: if the HPV test result was positive for types 16 or 18, it was recommended to undergo a colposcopy directly. If other high-risk HPV types were positive, a cytologic testing should be conducted. If the result was Atypical Squamous Cells of Undetermined Significance (ASCUS) or higher, colposcopy is recommended (19) (Figure 1).

### 2.3 Self-sampling collection

**Figure 2:**
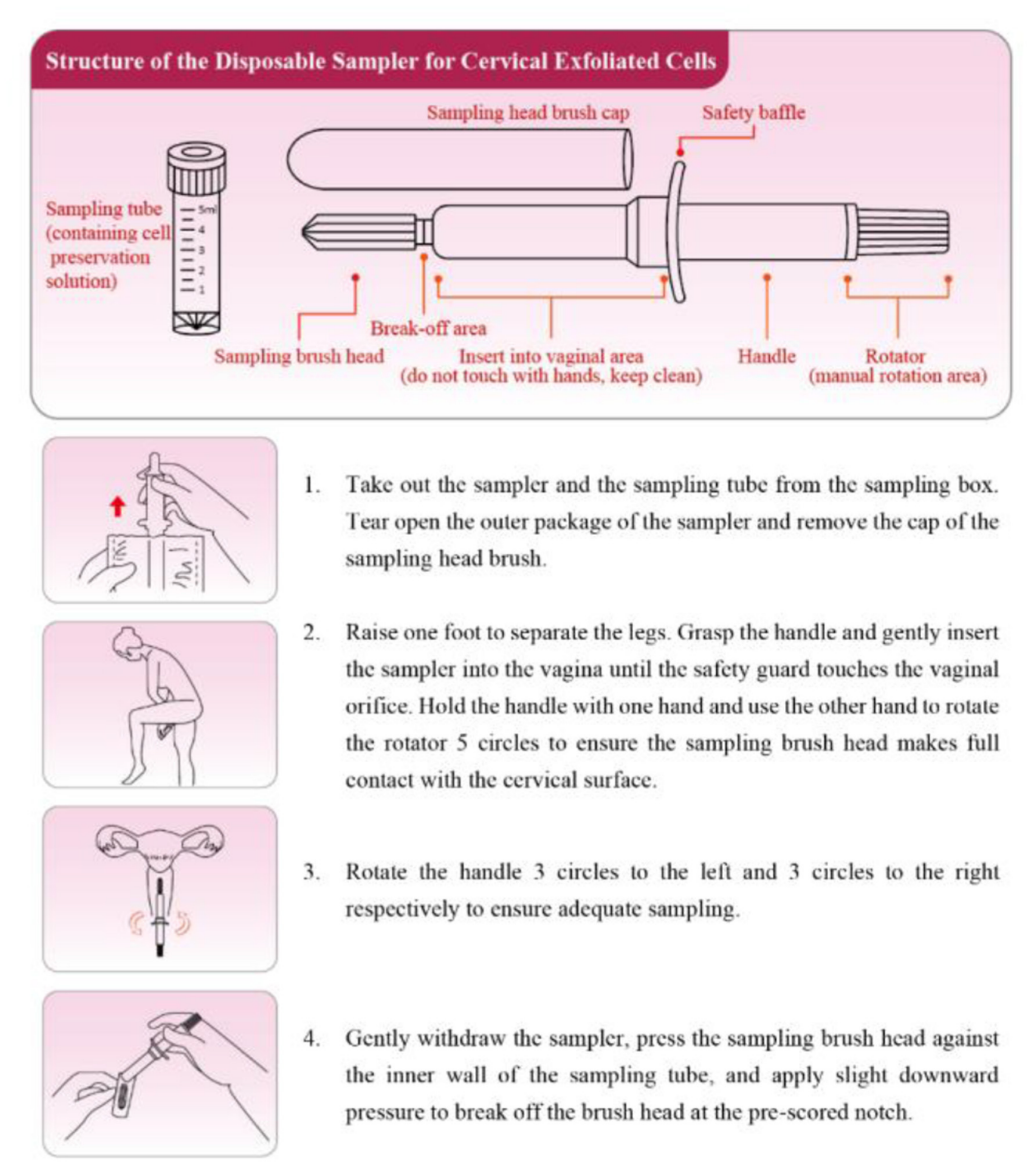
Operational guidelines of HPV self-sampling.

The participants were distributed self-sampling kits (Jiangsu Shuoshi Biotechnology Co., Ltd.; Sutai Medical Equipment Preparation No. 20180256) and placed in a half-squat position. The sampling brush was inserted ~5 cm into the vagina and touched the vaginal wall, rotated clockwise for 5 turns, removed, the brush head was placed on the blue solid transfer card in the test bottle, pressed and rubbed repeatedly until the card changed color. The brush head was discarded, and the sample bottle was retained. The operational guidelines of self-sampling were presented in Figure 2.

### 2.4 HPV testing

HPV typing and quantitative detection were performed via the BioPerfectus Multiple Real Time (BMRT) method (HPV typing and genetic testing kits; Jiangsu Shuoshi Biotechnology Co., Ltd.; National Medical Products Administration No. 20153400364). A real-time fluorescence quantitative PCR instrument (Applied Biosystems, 7500, USA) and HPV nucleic acid analysis quantitative analysis software (V1.0) were used to detect 21 types of HPV, including 14 high-risk HPV types: 16, 18, 31, 33, 52, 58, 35, 39, 45, 51, 56, 59, 68, and 66; 4 intermediate-risk HPV subtypes: 26, 53, 73, and 82; and 3 low-risk HPV subtypes: 6, 11, and 81.

The identification criteria for positive detection results of the HPV subtype were as follows: first, the experimental amplification curve of the HPV subtype was a typical S-shaped curve; when the CT value was less than the reference value of 36.7, a positive viral load was reported (virus copy number/10^4^ cells × number of cells in the sample).

In the analysis of HPV infection types, for HPV-positive participants, the infection with one of the 21 types was defined as single infection, and the simultaneous infection with multiple HPV subtypes was defined as multiple infection.

### 2.5 Cervical liquid-based cytologic testing

A total of 164 HPV-positive screeners were recalled for the ThinPrep^®^ Cytologic Test (TCT). Cervical exfoliative cells were collected by a specialized physician via a standardized cell-collecting brush that was rotated repeatedly over the cervix uteri and cervical canal for several turns, and samples were obtained from the brush head by repeated shaking in a vial of preservative solution (Hologic, USA), which was then retained for testing. Pathology results were independently analyzed and reported by at least two pathologists according to the The Bethesda System (TBS) classification, which included the following: 1. negative for intraepithelial lesion or malignancy (NILM); 2. epithelial cell abnormalities: (1) squamous epithelial cell abnormalities: atypical squamous cells of unknown significance (ASCUS), atypical squamous cells: cannot exclude high-grade squamous intraepithelial lesion (ASC-H), low-grade squamous intraepithelial lesion (LSIL), high-grade squamous intraepithelial lesion (HSIL), and cervical squamous cervical cancer (SCC); (2) glandular epithelial cell changes: atypical glandular epithelium, glandular carcinoma in situ and adenocarcinoma; and (3) other malignant tumors.

### 2.6 Colposcopy and histopathology

A total of 104 HPV-positive screeners were recalled for colposcopy. Colposcopy was performed by a specialized physician, who first observed the surface of the cervix after saline cleaning, then covered the surface of the cervix with cotton pads soaked in glacial acetic acid and observed the reaction after 1 min. Then, a cotton ball containing a tincture of iodine was applied to the surface of the cervix to observe the coloration, the cervical lesions or suspected lesions were biopsied according to the specific conditions, and cervical tube scraping was performed if necessary. The obtained tissues were fixed and submitted to two experienced pathologists for independent review. The histopathological results were based on the diagnostic criteria for cervical intraepithelial neoplasia and cervical cancer in the 10th edition of Pathology (20), categorized as no obvious abnormality, cervicitis, LSIL, HSIL, and invasive carcinoma.

### 2.7 Statistical analysis

SPSS 21.0 statistical software was used for statistical processing. The general data were statistically described, and the count data were expressed as %. The chi-square test was used for single-factor analysis of the relevant influencing factors. The logistic regression model and decision tree model were used for multifactorial analysis. The normality of the measurement data was tested via the Shapiro-Wilk test, and the results are expressed as χ^2^ ± s. The viral load was expressed as the median and was statistically analyzed via the Wilcoxon rank sum test. *P* < 0.05 indicated statistical significance.

## 3 Results

### 3.1 Demographic characteristics of the self-sampling screening population

In this study, a total of 1,228 women were tested via self-sampling for HPV types and quantity. The mean age of the screening group was 43.65 ± 10.23 years, with the majority of women aged 36–50 years. The mean height was 160.98 ± 4.75 cm, and the mean weight was 61.92 ± 10.49 kg. A total of 33.71% (414/1,228) of women in the surveyed population lacked knowledge about cervical cancer screening, with a predominance of those who had lower senior high school education, earned < 5,000 a month, were married, had more than 1 pregnancy, had not been tested for HPV before, had not been immunized against HPV and did not know about HPV self-sampling (Table 1).

**Table 1:** Characteristics of people with different levels of cognition.

**Characteristics**	**Total**	**Awareness of cervical cancer screening**		***χ^2^***	***P***
		**Didn't know (*****n*** = **414)**	**Knew (*****n*** = **814)**		
Age (year)				30.109	0.001
21–35	301	78	223		
36–50	594	185	409		
51–65	333	151	182		
Education				133.683	0.001
Junior and below	482	249	233		
Senior	369	110	259		
College and above	377	55	322		
Economic level (monthly income/yuan)				52.228	0.001
≤ 2,000	402	181	221		
2,001–4,999	518	174	344		
≥5,000	308	59	249		
Marital history				2.210	0.137
Unmarried	367	135	232		
Married	861	279	582		
Number of pregnancies				21.125	0.001
0	95	51	44		
1	150	57	93		
>1	983	306	677		
Number of deliveries				6.470	0.039
0	533	182	351		
1	227	61	166		
>1	468	171	297		
Had HPV testing				103.912	0.001
Yes	401	56	345		
No	827	358	469		
Got HPV vaccination				119.218	0.001
Yes	322	29	293		
No	906	385	521		
Understood self-sampling				9.694	0.002
Yes	34	3	31		
No	1,194	411	783		

### 3.2 Analysis of factors influencing awareness of cervical cancer screening

Analysis of variance revealed statistically significant differences in several aspects, such as age, educational level, economic level, number of pregnancies, history of previous testing, history of previous vaccinations, and knowledge of cervical cancer screening by self-sampling, between patients with and without cervical cancer screening awareness (χ^2^ = 30.109, 133.683, 52.228, 21.125, 103.912, 119.218, 9.694, and all *P* < 0.01; Table 1). Logistic regression analysis revealed that older age (OR = 1.233, *P* = 0.025) and lower educational level (OR = 0.448, *P* = 0.001) independently influenced HPV self-sampling and cervical cancer screening awareness in the screening population (Tables 2, 3).

**Table 2:** Assignments for logistic regression analysis.

**Characteristics**	**Description of variables**	**Description of the assignment**
Age	Categorical	21–35 years = “0”, 36–50 years = “1”, 51–65 years = “2”
Educational level	Categorical	Junior and below = “0”, senior = “1”, college and above = “2”
Economic level	Categorical	≤2,000 = “0”, 2,001–4,999 = “1”, ≥5,000 = “2”
Number of pregnancies	Categorical	0 = “0”, 1 = “1”, >1 = “2”

**Table 3:** Logistic regression analysis of factors influencing cervical cancer screening awareness.

**Characteristics**	***B* value**	**Wald**	***P***	**OR value**	**95% confidence interval**	
					**Upper bound**	**Lower bound**
Age	0.210	5.036	0.025	1.233	1.027	1.481
Educational level	−0.804	76.509	0.001	0.448	0.374	0.536
Economic level	−0.179	3.543	0.060	0.836	0.694	1.007
Number of pregnancies	0.458	0.917	0.338	1.580	0.619	4.032

### 3.3 Analysis of the distribution of positive HPV subtypes via self-sampling

Among the 1,228 self-sampling samples, 232 were positive for HPV infection, accounting for 18.89% (232/1,228) of the overall tested population, of which HPV26 and HPV73 were not detected. Among them, susceptible high-risk types accounted for 12.62%, general high-risk types accounted for 7.5%, medium-risk types accounted for 3.01%, low-risk types accounted for 2.93%. The high-risk types altogether accounted for 78.02% (181/232). Among the remaining 19 detected subtypes, the common subtypes of infection were HPV16, with a 2.61% (32/1,228) infection rate; HPV52, with a 2.44% (30/1,228) infection rate; HPV53, with a 2.28% (28/1,228) infection rate; HPV58, with a 2.28% (28/1,228) infection rate; and HPV81, with a 2.12% (26/1,228) infection rate (Table 4).

**Table 4:** Detection of subtypes via HPV self-sampling.

**Subtypes**	**Single infection (*n*)**	**Multiple infection (*n*)**	**Total (*n*/%)**
**Susceptible high-risk**			
16	13	19	32 (2.61)
52	20	10	30 (2.44)
58	13	15	28 (2.28)
68	13	12	25 (2.04)
51	12	10	22 (1.79)
39	6	12	18 (1.47)
**General high-risk**			
56	10	10	20 (1.63)
66	4	15	19 (1.55)
31	7	6	13 (1.06)
18	4	9	13 (1.06)
59	6	3	9 (0.73)
33	2	5	7 (0.57)
35	2	4	6 (0.49)
45	1	4	5 (0.41)
**Medium-risk**			
53	16	12	28 (2.28)
82	7	2	9 (0.73)
26	0	0	0 (0.00)
73	0	0	0 (0.00)
**Low-risk**			
81	9	17	26 (2.12)
6	4	4	8 (0.65)
11	0	2	2 (0.16)

### 3.4 HPV infection load in HPV self-sampling

This study also detected the viral loads of the HPV-positive samples: a viral load of HPV16 ≥10^4^ accounted for 31.25% (10/32) of those infected with HPV16, a viral load of HPV52 ≥10^4^ accounted for 43.33% (13/30), a viral load of HPV53 ≥10^4^ accounted for 28.57% (8/28), a viral load of HPV58 ≥10^4^ accounted for 28.57% (8/28), and a viral load of HPV81 ≥10^4^ accounted for 19.23% (5/26). The subtypes with viral loads ≥10^4^ accounting for more than 30% of the population were, in descending order, 68, 82, 52, 33, 56, 45, 31, 6, 51, 35, 16, and 18 (Table 5).

**Table 5:** HPV viral load of self-sampling.

**Subtypes**	**Viral load**		**Total (*n*)**
	≥**10**^4^ **(*****n*****)**	<**10**^4^ **(*****n*****)**	
**Susceptible high-risk**			
16	10	22	32
52	13	17	30
58	8	20	28
68	13	12	25
51	8	14	22
39	5	13	18
**General high-risk**			
56	8	12	20
66	5	14	19
31	5	8	13
18	4	9	13
59	2	7	9
33	3	4	7
35	2	4	6
45	2	3	5
**Medium-risk**			
53	8	20	28
82	4	5	9
26	0	0	0
73	0	0	0
**Low-risk**			
81	5	21	26
6	3	5	8
11	0	2	2

### 3.5 Follow-up analysis of self-sampling in HPV-positive patients

In this study, 232 positive patients were followed up, 164 were recalled for further TCT and 104 were recalled for further colposcopy and biopsy pathology. The TCT results revealed that 80.49% (132/164) of the women did not have significant abnormalities on cytologic screening, a total of 25 patients were found to have pathology results suggestive of LSIL and above, accounting for 24.04% (25/104) of the number of colposcopies performed (Table 6).

**Table 6:** Cytologic and pathologic follow-up results.

**Colposcopic follow-up results**	**Liquid-based cytology results**			
	**NILM (** ***n*** **)**	**ASC-US (** ***n*** **)**	**ASC-H (** ***n*** **)**	**LSIL (** ***n*** **)**
Normal and cervicitis	72	7	0	0
LSIL	5	4	1	3
HSIL	2	5	3	2
Without follow-up	53	4	0	2
Total	132	21	4	7

### 3.6 Analysis of HPV infection factors in self-sampling

HPV-positive samples obtained via self-sampling revealed that the factors associated with HPV infection included age at first sexual intercourse (χ^2^ = 6.530, *P* = 0.011), frequency of sexual intercourse (χ^2^ = 5.395, *P* = 0.020), number of deliveries (χ^2^ = 5.471, *P* = 0.019), washing after coitus (χ^2^ = 13.014, *P* = 0.001), condom use during intercourse (χ^2^ = 14.177, *P* = 0.001) and multiple sexual partners (χ^2^ = 5.308, *P* = 0.021; Table 7).

**Table 7:** Analysis of HPV infection factors.

**Factors**	**Positive (*n* = 232)**	**Negative (*n* = 996)**	**χ^2^**	***P***
Body mass index (BMI)			0.135	0.713
Normalcy	124	519		
Abnormality	108	477		
Age (year)			0.995	0.608
21–35	53	248		
36–50	119	475		
51–65	60	273		
Age at first sexual intercourse (y)			6.530	0.011
≤ 20	74	237		
>20	158	759		
Sexual frequency (times/week)			5.395	0.020
≤ 2	206	929		
>2	26	67		
Number of pregnancies			1.086	0.297
≤ 1	52	193		
>1	180	803		
Number of deliveries			5.471	0.019
≤ 1	128	632		
>1	104	364		
Smoking history			1.049	0.306
Yes	2	18		
No	230	978		
HPV vaccination			1.832	0.176
Yes	12	77		
No	220	919		
Postcoital cleansing			13.014	0.001
Frequently	18	172		
Occasionally or not	214	824		
Use condoms during intercourse			14.177	0.001
Frequently	81	484		
Occasionally or not	151	512		
Multiple sexual partners			5.308	0.021
Yes	6	8		
No	226	988		

### 3.7 Multifactorial analysis of HPV infection factors in self-sampling

**Figure 3:**
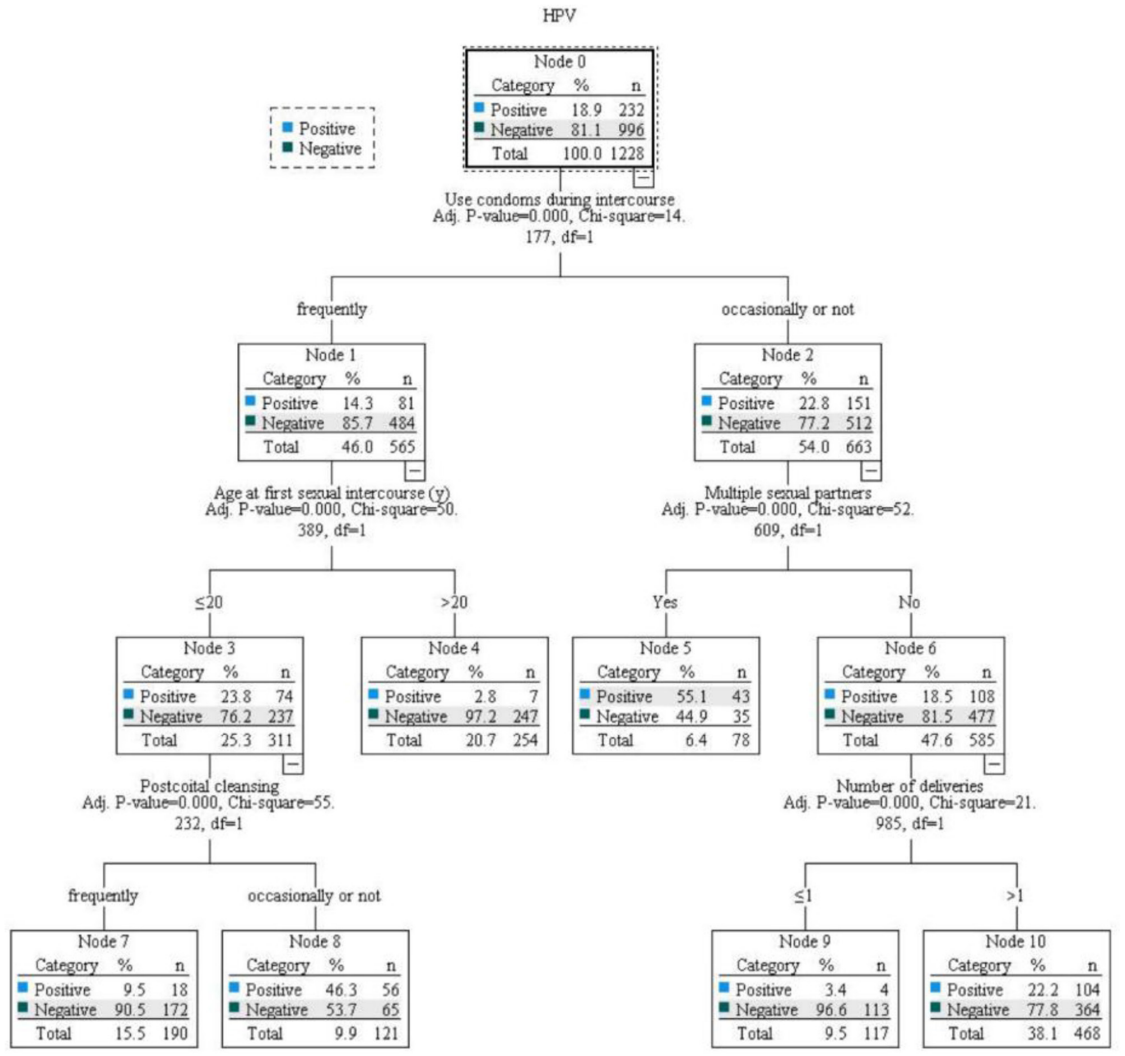
Analysis of HPV infection factors by decision tree model.

Logistic regression analysis of all the influencing factors revealed that age at first sex ≤ 20 years, having >1 deliveries, sex frequency >2 times/week, having multiple sexual partners were risk factors for the occurrence of HPV infection (OR > 1, *P* < 0.05), and frequently washing after coitus and frequently using condoms during coitus were protective factors (OR < 1, *P* < 0.05; Table 8, Figure 3).

**Table 8:** Logistic regression analysis of HPV infection.

**Characteristics**	***B* value**	**Wald**	***P***	**OR value**	**95% confidence interval**	
					**Upper bound**	**Lower bound**
Body mass index (BMI)	0.052	0.109	0.741	1.054	0.773	1.435
Age	−0.064	0.265	0.607	0.938	0.734	1.198
Age at sexual intercourse ≤ 20 years	0.435	6.625	0.010	1.544	1.109	2.150
Sexual frequency >2 times/week	0.625	5.860	0.015	1.868	1.126	3.097
Pregnancy >1	0.084	1.761	0.184	1.088	0.961	1.231
Deliveries >1	0.530	8.810	0.003	1.699	1.197	2.410
Smoking	−0.866	1.287	0.257	0.421	0.094	1.878
HPV vaccination	−0.356	1.174	0.278	0.700	0.368	1.334
Wash frequently after intercourse	−0.984	13.289	0.001	0.374	0.220	0.634
Use condom frequently during intercourse	−0.670	14.682	0.001	0.512	0.363	0.721
Multiple sexual partners	1.503	6.318	0.012	4.496	1.393	14.519

### 3.8 Analysis of experience with HPV self-sampling from screening populations

The analysis of the acceptability of the self-sampling procedure revealed that 62.54% of the women took less time for self-sampling; 84.12% of the women considered self-sampling easy to perform; 70.85% of the women felt the self-sampling process comfortable and without significant pain; 89.17% of the women did not have any significant bleeding after the sampling; and 84.20% of the women were willing to take HPV self-sampling again in the future (Table 9).

**Table 9:** HPV self-sampling experience survey of screening populations.

**Evaluation of self-sampling**	**Number of cases (*n*)**	**Percentage (%)**
**Time-consuming**		
Fast (< 5 min)	768	62.54
General (5–10 min)	331	26.95
Slow (>10 min)	129	10.50
**Operationalization**		
Easy	1,033	84.12
Complicated	195	15.88
**Experience feeling**		
Comfortable and painless	870	70.85
Discomfort	335	27.28
Excruciating	23	1.87
**Bleeding**		
Yes	133	10.83
No	1,095	89.17
**Willingness to reaccept**		
Yes	1,034	84.20
No	194	15.80

## 4 Discussion

This study revealed that the overall HPV infection rate of female residents in the community of Zhengzhou city, Henan Province, was 18.89%, with more positive cases of high-risk infections and single-subtype infections. HPV 16 accounted for the highest percentage, followed by HPV 52, 58, and 53. HPV type 81 was detected most frequently among the low-risk HPV types of infections associated with genital wart lesions, which was generally consistent with the results of a large-sample retrospective study of 240,000 people in 29 provinces, cities and autonomous regions of China, which revealed that the overall HPV infection rate in China's female population was 15.3%-24.4% (21), with the highest positive rates of HPV 52, 16, and 53 and obvious geographic differences. Women in this region are still at a high level of HPV infection, with HPV 16, 52, 58, and 53 being the most common, and the situation of screening, prevention, and control of HPV infection is still serious. Summarizing the characteristics of HPV infection in women in this region can provide a reference for the prevention and control of cervical cancer.

This study used a typed and quantitative assay, which was first proposed by FCLSCs in 2018, in which the load is indicative of the detection of high-grade cervical lesions (22, 23). The significance of detecting the load of infection in women in the region was analyzed for timely screening of high-grade cervical lesions in the region. The results of this study revealed that the subtypes with loads >10^4^ in HPV infection-positive cases were mostly concentrated in the HPV16, 52 and 68 subtypes, followed by the HPV58, 51, 56, and 53 subtypes. The difference in the distribution of loads among the high-risk types 10^4^ was not significant, but the proportion of susceptible high-risk types with loads >10^4^ was ~50%. This finding indicates that the susceptible high-risk types not only have a high infection rate but also have a high viral load after infection among female community residents in Zhengzhou city, Henan Province. In addition, many reports have confirmed that a load of 10^4^ is instructive for ASCUS triage and suggestive of cervical lesions (24, 25).

This study revealed that female community residents in Zhengzhou city, Henan Province, lacked effective knowledge of cervical cancer screening, and very few women were aware of HPV self-sampling, with older age and low literacy levels as independent influences on their low level of knowledge. Compared with women over 50 years of age, those under 50 years of age are more likely to have a certain degree of social cognition and health awareness, a relatively greater level of knowledge of HPV, familiarity with cervical cancer, and self-sampling. This may be because older women with lower literacy levels retain traditional Chinese ideology, have lower acceptance of gynecological examinations, and are less motivated to participate in screening. The aging of China's population has intensified, and this group of people has been the key target of cervical cancer screening; thus, in the community popularization of science and clinical consultation, we should focus on older women with low literacy levels to carry out easy-to-understand cervical cancer screening and the advantages of HPV self-sampling tests and carry out incentives for screening if necessary to increase the acceptance of this group of people in HPV self-sampling tests. Thus, strengthening the knowledge of cervical cancer among women can improve the understanding of cervical cancer and increase the awareness of women. Enhancing women's knowledge of cervical cancer can increase the motivation of the population to participate in screening, thus expanding the coverage of cervical cancer screening in the population.

Among the 232 patients with positive HPV infection, some irresistible external factors and patients' personal will were excluded. Among the 164 patients who could receive normal follow-up, 12 patients had high-level cervical lesions, accounting for a ratio of 7.32%, among which 9 patients had HPV loads higher than 10^4^, accounting for 75% of high-level cervical lesions. These findings indicated that only a small proportion of positive patients can further develop high-grade disease. It takes a long time to progress to cervical cancer, so the popularization of convenient and effective cervical cancer screening methods plays a key role in the prevention and treatment of cervical cancer and greatly reduces the risk and chances of progressing to cervical cancer (26). These findings also suggest that improve the detection rate of high-grade lesions and the reduction in missed detections achieved by testing the viral load are applicable in Henan province.

This study revealed that age at first sexual intercourse, frequency of sexual intercourse, number of births, number of sexual partners, washing after intercourse and use of condoms during intercourse were factors associated with HPV infection in female residents of the community of Zhengzhou city, Henan Province. Among them, young age at first sexual intercourse, a high number of births, a high frequency of sexual intercourse, and having multiple sexual partners were risk factors for the occurrence of HPV infection. HPV infection is transmitted mainly by sexual contact, and women who have their first sexual intercourse at an early age, inappropriate methods of sexual contact, and physiological factors such as cervical inflammation can increase the risk of HPV infection (27). Although it has also been reported that the degree of cervical pathology is not related to the age of initial sexual intercourse, it may be related to geographic location (25). Women who are sexually active or have multiple male partners can have unhealthy sexual behaviors that put the cervix at risk for repeated exposure to infections and pathogenic factors. Multiple vaginal births in women can cause localized tissue damage to the cervix, resulting in multiple occurrences of cellular oxidative stress that may cause damage and viral integration at the DNA level (28), leading to HPV susceptibility. In this study, frequent washing after intercourse and frequent condom use during intercourse were found to be protective factors against HPV infection. Cleaning the genitals after sexual intercourse reduces genital and urinary tract infections, thus reducing the risk of HPV infection (29, 30). Unprotected intercourse is a high-risk factor for HPV infection (31). At present, there are few studies on cleaning after intercourse as an influencing factor for maintaining hygienic intercourse environments to ward off HPV infection, but this study suggested that taking contraceptive measures, such as using condoms to avoid the growth of pathogens due to sexual contact, can reduce the chance of HPV infection.

The difference between smoking status and HPV vaccination status for HPV infection in this study were not statistically significant, which are inconsistent with some studies. It currently considers that vaccination can reduce the rate of HPV infection and that smoking can increase the risk of cervical disease (25, 27). The reasons may be that the questionnaire in this study did not add a female passive smoking inquiry, and the overall HPV vaccination rate was considered geographically specific, especially in the populous province of Henan, which is very low. The residents' knowledge of HPV vaccination needs to be improved, and bivalent, quadrivalent and nine-valent vaccines are not able to protect against all high-risk types of HPV infection. Analyzing the influencing factors of HPV infection can provide reliable data for the primary prevention of cervical cancer in this region. Strengthening the popularization of science for key populations and focusing on the popularization of knowledge about healthy sexual behaviors and appropriate pregnancy and childbirth can effectively reduce the level of HPV infection among women in Henan Province.

There are already some reports on the usage experience of HPV self-sampling among women participating in cervical cancer screening (32, 33), but our study is the first investigation of the usage experience of HPV self-sampling in the populous province of Henan, and the advantages and disadvantages of usage experience affect women's acceptance of this new screening mode. This study revealed that 84.2% of women tend to choose self-sampling screening again in the future and that HPV self-sampling testing had high acceptance in the population. The survey results revealed that 84.12% of women considered self-sampling easy, 70.85% of women felt comfortable during self-sampling, and 89.17% of women experienced no discomfort, such as bleeding, after self-sampling, which were consistent with the results of the study conducted by Zhao et al. (32, 33). HPV self-sampling tests can avoid the embarrassment caused by physicians using specula for gynecological examinations and reduce the degree of resistance caused by contacting the private parts of women; thus, it is more advantageous to promote HPV self-sampling test as a mean of cervical cancer screening than medical sampling in Henan province.

Primary prevention methods for cervical cancer include HPV vaccination and the popularization of cervical cancer knowledge. The results of this study revealed that the incidence of HPV infection in female residents in the community of Zhengzhou city was high, the common subtypes of infection were all high-risk oncogenic subtypes, and the management of high-risk groups of cervical lesions should be strengthened during subsequent triage. In promoting primary prevention of cervical cancer, emphasis should be placed on the popularization of condom use, healthy sexual behavior, and moderate pregnancy and childbirth to reduce the chance of HPV infection. The promotion of appropriate cervical cancer screening programs requires comprehensive consideration of various social, economic, and environmental factors. HPV self-sampling was named one of the “Top 10 global medical innovations that will influence the future” in 2017 (34), and the guidelines released by the WHO on July 6, 2021, mention self-sampling as a screening tool. However, many current studies have focused on the consistency and feasibility of self-sampling vs. traditional medical sampling and the sensitivity characteristics of the quantitative nucleic acid typing method for HPV testing and other testing methods. Studies on the usage experience of HPV self-sampling have not been comprehensive or complete. This study revealed that the cervical cancer screening model based on the HPV self-sampling test had good results, high acceptance in the population, and good compliance with follow-up, which can be promoted to improve the coverage of cervical cancer screening. Although this study did not include the verification of consistency with medical sampling, the associations with high-grade cervical lesions, and the cost-benefit analysis, and there was a lack of specific analysis of non-participants and those who were lost to follow-up in the initial research design, the subsequent expansion of sample data will conduct specific analyses of the above issues for different populations in Henan, and compare the epidemiological background of those with high-grade and low-grade subtypes. This study found that wash frequently after intercourse and use condom frequently during intercourse are obstacles to HPV infection, which can also prevent the occurrence of virus reinfection. Self-sampling also effectively avoids the possible infections that may occur during the hospital examination process. In our clinical experience, good living habits, rest and diet rules, moderate exercise, improve immunity are conducive to HPV infection and reinfection. Moreover, HPV typing and quantitative testing can reveal the subtype and viral load of HPV infection, which provides guidance for the triage of HPV-positive populations (35), and HPV self-sampling testing is more advantageous in addressing tense situations, such as the epidemic of coronavirus disease, and is expected to be promoted and widely applied nationwide for a long period of time.

## 5 Conclusion

This study demonstrates that the use of HPV typing and quantitative detection methods is a good approach worthy of reference among the female residents in Zhengzhou City, Henan Province. Paying attention to the age at first sexual intercourse, sexual frequency, parity, the number of sexual partners, cleaning behavior after sexual intercourse, and condom use during sexual intercourse can effectively reduce the risk of HPV infection. This study show that HPV self-sampling detection offers a good experience and has high acceptance, and can be popularized and applied in cervical cancer screening in Henan Province, which provides a new approach to reducing the incidence of cervical cancer.

## Data Availability

The datasets presented in this article are not readily available due to privacy or ethical restrictions. Requests to access the datasets should be directed to zheyang0102@126.com.
