# Supplementary material for: A study of HPV self-sampling by female residents in communities of Zhengzhou, Henan Province, China: a cross-sectional observational study
Source: Front Public Health. 2025 Jul 1;13:1557678. doi: 10.3389/fpubh.2025.1557678 (PMC12259585; doi:10.3389/fpubh.2025.1557678)
Supplement: Supplementary file 1 [file Presentation_1.pdf]

HPV Self-sampling Questionnaire

一、Essential Information

Name：__________ Age：_____y Height：________cm Weight：______kg

Mobile Phone Numbers：___________________ID Numbers：______________________

二、Investigation of High Risk Factors

1. Age at first sexual intercourse _y
2. Sexual frequency ___(times/week)
3. Number of pregnancies ___ times
4. Number of deliveries ___ times
5. Smoking history ☐Yes ☐No
6. Already menopausal ☐Yes ☐No
7. Postcoital cleansing

☐Yes ☐No

8. Taking regular exercise

☐Yes ☐No

9. History of recurrent vaginitis

☐Yes ☐No

10. History of recurrent cervicitis

☐Yes ☐No

11. Multiple sexual partners

☐No ☐Yes ☐Unknow

12. History of HPV vaccination

☐No ☐Yes

13. History of HPV infection

☐No ☐Yes，____________

14. History of gynecological surgery

☐No ☐Yes，____________

15. Familial history of cancer

☐No ☐Yes，____________

16. Use condoms during intercourse

☐Yes ☐No

17. Understanding of cervical cancer screening

☐Don't understand ☐General

☐Understand very well

18. Educational level

☐Primary and below ☐Junior

☐Senior ☐College and above

19. Economic level（monthly income/yuan）

☐≤2000 yuan ☐2001-4999 yuan

☐≥5000 yuan

三. Investigation on acceptance of HPV self-sampling

1. Watch HPV self-sampling video

☐Yes ☐No

2. Operationalization

☐Easy ☐General ☐Complicated

3. Experience feeling

☐Comfortable and painless

☐Discomfort

☐Excruciating

4. Time-consuming

☐Fast（＜5min）

☐General（5-10min）

☐Slow（＞10min）

5. Bleeding after sampling

☐No bleeding ☐Bleeding spotting

☐Slight bleeding ☐Excessive bleeding

6. The advantages of choosing self-sampling HPV testing

☐Reduce the time to go to the hospital and the cost of transportation

☐The collection method is simple and convenient

（According to the law, the personal information in the questionnaire is strictly confidential and will not disclose any of your personal information.）

Sign Date

☐More protection of personal privacy

☐Others：_____________

7. Have you encountered the following difficulties：

☐There are doubts about the sampling method, and the sampling operation is not smooth.

☐Feeling pain and discomfort when sampling, and even bleeding.

☐Doubt about the accuracy of sample detection.

☐Others：____________

8. Willingness to reaccept in the future（ ）

☐Yes ☐General ☐No

（According to the law, the personal information in the questionnaire is strictly confidential and will not disclose any of your personal information.）

Sign Date
